# Toxicology and efficacy of tumor-targeting *Salmonella typhimurium* A1-R compared to VNP 20009 in a syngeneic mouse tumor model in immunocompetent mice

**DOI:** 10.18632/oncotarget.17605

**Published:** 2017-05-03

**Authors:** Yong Zhang, Wenluo Cao, Makoto Toneri, Nan Zhang, Tasuku Kiyuna, Takashi Murakami, Scott D. Nelson, Sarah M. Dry, Yunfeng Li, Shukuan Li, Xiaoen Wang, Huaiyu Ma, Arun S. Singh, Fritz C. Eilber, Robert M. Hoffman, Ming Zhao

**Affiliations:** ^1^ AntiCancer, Inc., San Diego, California, USA; ^2^ Department of Surgery, University of California, San Diego, California, USA; ^3^ Department of Pathology, University of California, Los Angeles, California, USA; ^4^ Department of Hematology/Oncology, University of California, Los Angeles, California, USA; ^5^ Division of Surgical Oncology, University of California, Los Angeles, California, USA

**Keywords:** Salmonella typhimurium A1-R, VNP20009, toxicity, tumor targeting, biodistribution

## Abstract

*Salmonella typhimurium* A1-R (*S. typhimurium* A1-R) attenuated by leu and arg auxotrophy has been shown to target multiple types of cancer in mouse models. In the present study, toxicologic and biodistribution studies of tumor-targeting *S. typhimurium* A1-R and *S. typhimurium* VNP20009 (VNP 20009) were performed in a syngeneic tumor model growing in immunocompetent BALB/c mice. Single or multiple doses of *S. typhimurium* A1-R of 2.5 × 10^5^ and 5 × 10^5^ were tolerated. A single dose of 1 × 10^6^ resulted in mouse death. *S. typhimurium* A1-R (5 × 10^5^ CFU) was eliminated from the circulation, liver and spleen approximately 3-5 days after bacterial administration via the tail vein, but remained in the tumor in high amounts. *S. typhimurium* A1-R was cleared from other organs much more rapidly. *S. typhimurium* A1-R and VNP 20009 toxicity to the spleen and liver was minimal. *S. typhimurium* A1-R showed higher selective targeting to the necrotic areas of the tumors than VNP20009. *S. typhimurium* A1-R inhibited the growth of CT26 colon carcinoma to a greater extent at the same dose of VNP20009. In conclusion, we have determined a safe dose and schedule of *S. typhimurium* A1-R administration in BALB/c mice, which is also efficacious against tumor growth. The results of the present report indicate similar toxicity of *S. typhimurium* A1-R and VNP20009, but greater antitumor efficacy of *S. typhimurium* A1-R in an immunocompetent animal. Since VNP2009 has already proven safe in a Phase I clinical trial, the present results indicate the high clinical potential of *S. typhimurium* A1-R.

## INTRODUCTION

For more than 200 years, there have been reports that cancer patients went into remission after recovering from bacterial infections [1]. From the late 19th to early 20th century, William Coley, an American physician, treated cancer patients with both live and heat-killed bacteria such as *Streptococcus pyogenes* and *Serratia marcescens* as first-line therapy for sarcoma and lymphoma. The combination of Coley's heat-killed bacteria was referred to as “Coley's toxins, which remained in clinical use for sarcoma patients until 1963 [1]. Since 1976, BCG (Bacillus Calmette-Guerin) bacteria have been used to treat superficial bladder cancer [2], which is still in clinical use [3].

There has been a great resurgence in preclinical research on bacterial therapy of cancer in the last 20 years [4]. *Salmonella* species are the most extensively studied bacteria in the field of tumor targeting. They are Gram-negative facultative-anaerobic bacteria that can grow and replicate inside host cells. Some *Salmonella* strains can preferentially colonize solid tumors [5–8].

*Salmonella typhimurium* VNP20009 (VNP20009) is attenuated with a lipid A–mutation (*msbB*), purine auxotrophy (*purI*) and amino acid autotrophy [9]. In a Phase I clinical trial on patients with metastatic melanoma and renal-cell carcinoma in the United States, VNP20009 was not toxic but poorly colonized the patients’ tumors, perhaps because it was over attenuated [10]. At the highest tolerated dose, some tumor colonization was observed [10].

Another strain of *S. typhimurium* A1-R, auxotrophic for leu-arg has been developed by our laboratory. *S. typhimurium* A1-R was effective against primary and metastatic tumors as monotherapy in nude mouse models of major cancers, including prostate [8, 11], breast [12–14], lung [15, 16], pancreatic [17–21], ovarian [22, 23] stomach [24], and cervical cancer [25]. In addition, *S. typhimurium* A1-R was effective against patient-derived orthotopic models (PDOX) of pancreatic cancer [17, 21], sarcoma [26–28] and melanoma [29–32].

Tumors with a high degree of vascularity were more sensitive to *S. typhimurium* A1-R, and vascular destruction appears to play a role in *S. typhimurium* A1-R antitumor efficacy [16].

In the present report, we compare efficacy, tumor targeting and toxicity of *S. typhimurium* A1-R and VNP20009, since VNP20009 has been tested to be safe in a Phase I clinical trial [10].

## RESULTS AND DISCUSSION

### Correlation between optical sensitivity and colony counting to quantitate bacteria

**Figure 1:**
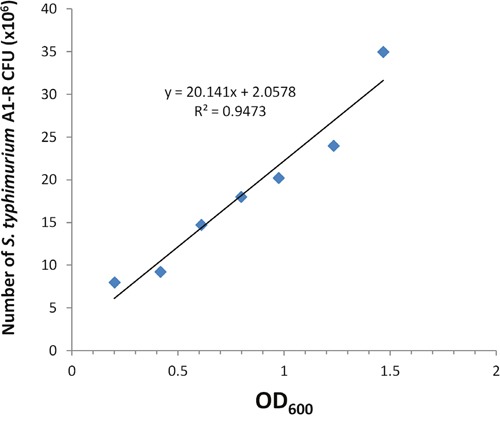
Comparison of OD600 and colony counting to quantify bacteria GFP-expressing *S. typhimurium* A1-R-GFP was grown overnight in LB medium and then diluted 1:10 in LB medium. Bacteria were harvested at late-log phase, washed with PBS, and then diluted in PBS. OD_600_ measurements were performed on a spectrophotometer at dilutions of 10×. Each dilution analysis of bacteria in PBS was plated and cultured overnight in LB agar and the number of resulting bacterial colonies was counted. Regression line demonstrates the correlation between colony-forming units with (CFU) bacteria and OD_600_.

In order to determine viable bacterial counts, we analyzed the relationship between the OD_600_ value and bacteria colony forming units (CFU) on LB agar. A linear relationship between OD_600_ and CFU was observed for *S. typhimurium* A1-R: Y = 20.141x + 2.0578, R^2^ = 0.9473) (Figure 1).

### Effect of a single-dose *S. typhimurium* A1-R on body weight of non-tumor-bearing BALB/c mice

**Figure 2:**
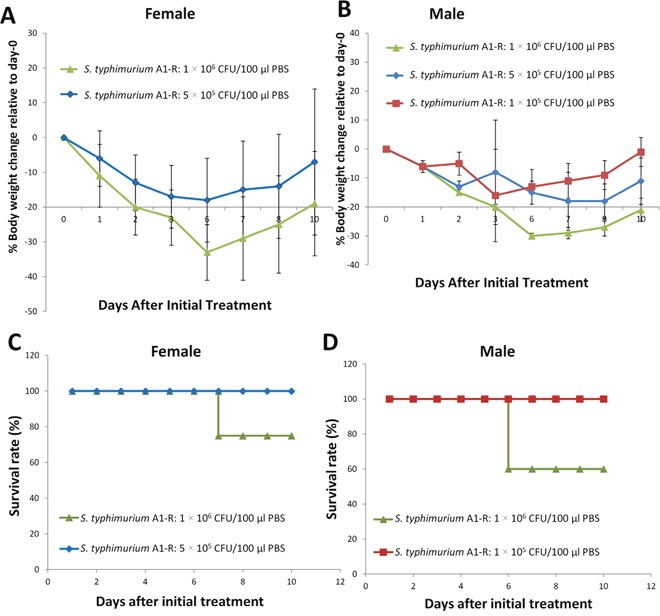
Effect of a single dose of *S. typhimurium* A1-R on body weight of non-tumor-bearing mice Normal BALB/c mice, aged 6 weeks, were injected with *S. typhimurium* A1-R at different doses in 100 μl PBS into the tail vein. Body weight was measured using an electronic scale. **(A)** Body weight curve for male female (n = 5). **(B)** Body weight curve for male mice. n = 5 mice for each group. **(C)** Survival time curve for female mice (n = 5). **(D)** Survival time curve for male mice. n = 5 mice for each group.

Six- to 8-week-old BALB/c mice without tumors, with an average body weight 20 to 22 gram, were used in the study. A single-dose of *S. typhimurium* A1-R at 1 × 10^5^ - 5 × 10^5^ CFU/mouse was administred i.v. Acute weight loss of 10% to 20% from day 0 was observed in all cohorts. However, body weight began recover by day 6. However, 1 × 10^6^ CFU/mouse did cause severe weight loss (Figure 2A, 2B). No signs of delayed toxicity were observed. *S. typhimurium* A1-R doses at 1 × 10^5^ and 5 × 10^5^ CFU did not cause mouse death, but 1 × 10^6^ CFU did (Figure 2C, 2D).

### Effect of a single dose of *S. typhimurium* A1-R on body weight of tumor-bearing BALB/C mice

**Figure 3:**
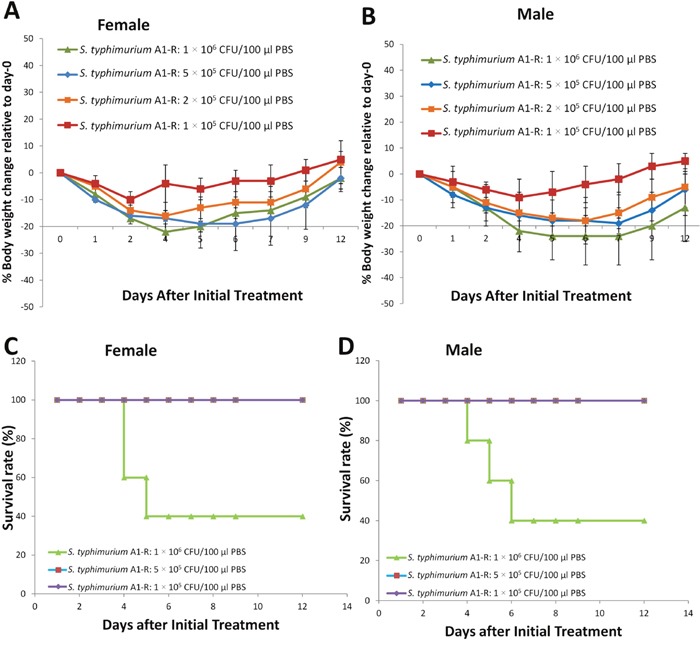
Effect of a single dose of *S. typhimurium* A1-R on body weight of tumor-bearing mice BALB/c mice bearing CT26 tumors with a tumor volume of 100 mm^3^ were treated with *S. typhimurium* A1-R at different doses in 100 μl PBS into the tail vein. Body weight was measured using an electronic scale. **(A)** Body weight curve for female mice (n = 5). **(B)** Body weight curve for male mice. n = 5 mice for each group. **(C)** Survival curve for female mice (n = 5). **(D)** Survival curve for male mice. n = 5 mice for each group.

Single doses of 1 × 10^5^, 5 × 10^5^ and 1 × 10^6^ were given to BALB/c mice bearing subcutaneous CT26 murine colon tumors. The body weight loss in the tumor-bearing mice was less than in the normal mice. The tumor-bearing mice which were given the 1 × 10^6^ CFU dose of *S. typhimurium* A1-R rapidly recovered unlike the non-tumor bearing mice (Figure 3A, 3B). However, although no deaths were observed at the two lower doses, the 1 × 10^6^ CFU dose caused death (Figure 3C, 3D).

### Effect of multiple doses of *S. typhimurium* A1-R on the body weight of tumor-bearing mice

**Figure 4:**
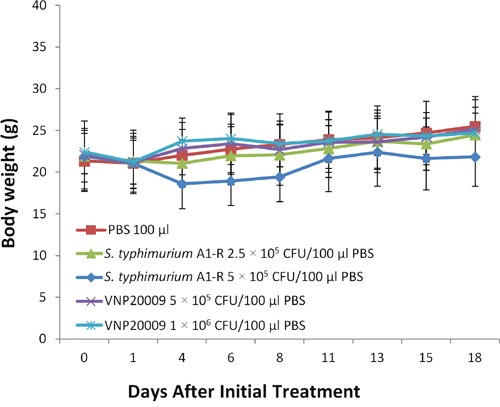
Effect of multiple doses of *S. typhimurium* A1-R and *S. typhimurium* VNP20009 on body weight of tumor-bearing mice Mice were treated with 2.5 × 10^5^ CFU, and 5 × 10^5^ CFU *S. typhimurium* A1-R weekly or 5 × 10^5^ and 1 × 10^6^ CFU VNP20009 weekly × 3. All treatments were administered i.v.

Based on the single-dose toxicologic results, we also assessed the toxicity of multiple doses of *S. typhimurium* A1-R compared with PBS administered to tumor-bearing mice (Figure 4). Weekly doses of *S. typhimurium* A1-R at 2.5, or 5 × 10^5^ CFU/mouse, were administered for 3 weeks. The 2.5 × 10^5^ CFU weekly doses did not cause noticeable weight loss. The 5 × 10^5^ CFU/mouse caused a body weight loss by day-4 which was recovered by day-11. Weekly doses of VNP20009 at 5 × 10^5^ or 1 × 106 CFU/mouse were administered for 3 weeks without noticeable weight loss.

### Clearance of *S. typhimurium* A1-R from the circulation

**Figure 5:**
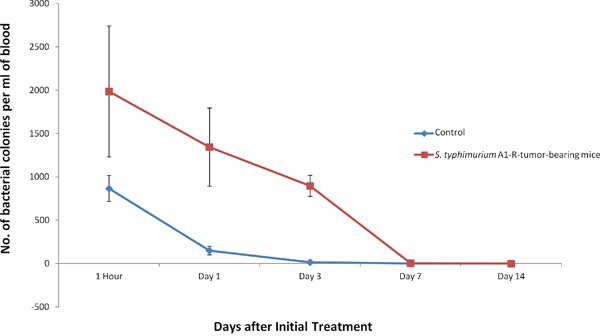
Bacteria clearance from blood circulation *S. typhimurium* A1-R (5 × 10^5^ CFU/100 μl PBS/mouse) were injected into non-tumor-bearing mice or CT26 tumor-bearing BALB/c mice. Bacteria in 100 μl of blood were counted after overnight culture in LB agar. Values in both graphs represent the mean (with standard deviation) of at least three mice per time point.

To quantitatively assess the clearance of bacteria from the circulation, GFP-labeled *S. typhimurium* A1-R (*S. typhimurium* A1-R-GFP) was intravenously injected into non-tumor-bearing and tumor-bearing BALB/c mice. *S. typhimurium* A1-R-GFP in the blood was collected, cultured, and counted at various times after injection using three mice per time point. Greater than 95% of the bacteria were cleared from the circulation within 1 day in non-tumor-bearing mice, and the number of CFU fell to barely detectable levels over the next 7 days (Figure 5). Bacteria in tumor-bearing mice were cleared more slowly (Figure 5).

### Biodistribution of bacteria

**Figure 6:**
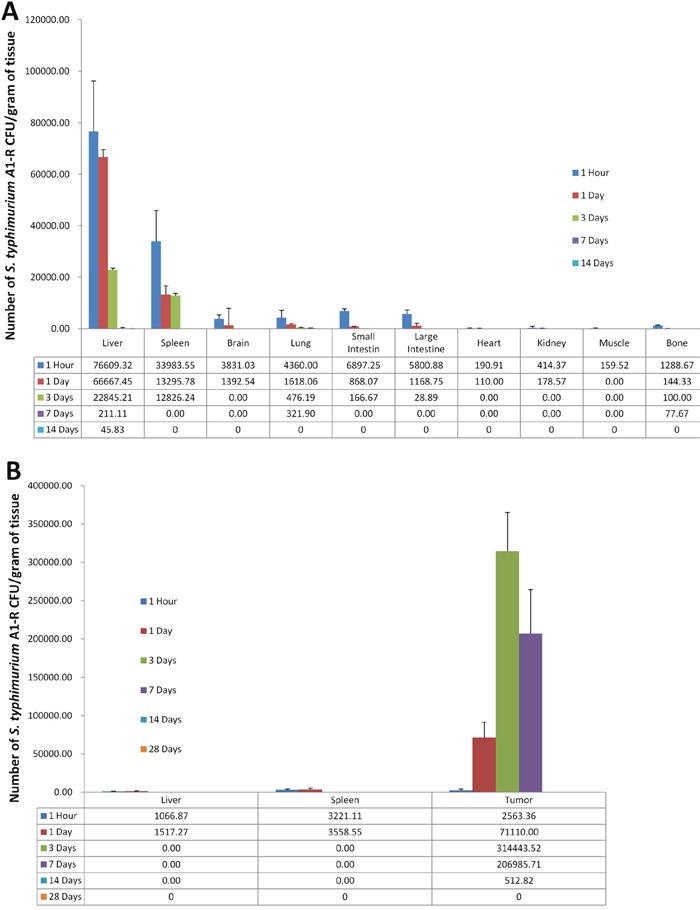
Distribution of *S. typhimurium* A1-R *S. typhimurium* A1-R (5 × 10^5^ CFU/100 μl PBS/mouse) were injected into normal **(A)** or CT26 tumor-bearing BALB/c mice **(B)**. Tissues were removed at time points indicated and homogenized. Homogenates from isolated from the tumors and organs were cultured in LB agar. At least three mice were evaluated per time point.

To determine the distribution of bacteria, normal and tumor-bearing BALB/c mice were injected with *S. typhimurium* A1-R GFP. Organs and tumors were collected, homogenized and *S. typhimurium* A1-R was cultured on LB agar. The number of CFU was counted from organs starting 1 hour after injection. Bacteria colonies from organs and tumors were subsequently observed by fluorescence microscopy. In non-tumor-bearing mice, significant *S. typhimurium* A1-R-GFP colonies from organs were present at 1 h after injection, but were undetectable 1week later. The largest amount of *S. typhimurium* A1-R-GFP was present in the liver and spleen, but was cleared by 7 days. The other organs were cleared by 3 days (Figure 6A).

In the tumor-bearing mice, *S. typhimurium* A1-R-GFP in tumors survived much longer than in the normal organs (Figure 6B).

**Figure 7:**
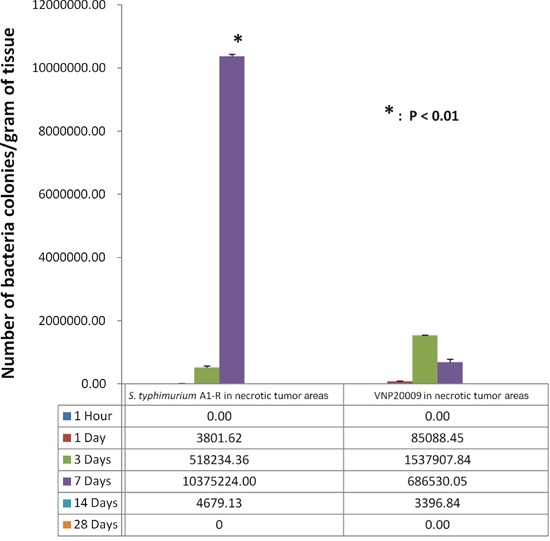
*S. typhimurium* A1-R and VNP20009 were isolated from the necrotic areas of tumors at various time points

**Figure 8:**
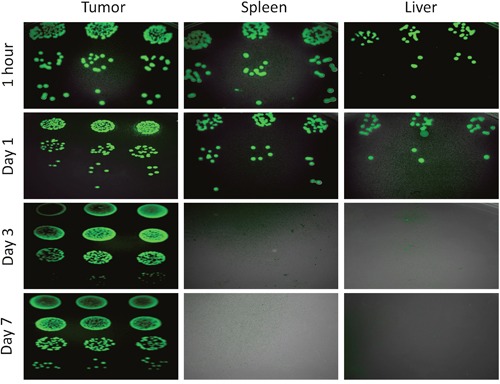
Fluorescence images of *S. typhimurium* A1-R-GFP isolated from the tumor, spleen and liver at various time points on LB agar

*S. typhimurium* A1-R-GFP and VNP20009-RFP were isolated from the viable and necrotic tumor regions and cultured in LB agar. *S. typhimurium* A1-R survived in tumor necrotic regions more than VNP20009 at day-7 when the bacteria were administered i.v. (p<0.01) (Figure 7). FP Fluorescent colonies of *S. typhimurium* A1-R-GFP isolated from the tumor, spleen and liver at various time points were imaged. Fluorescent colonies are seen derived from the tumor at all time points from 1 hour to day 7. Fluorescent colonies can be seen isolated from the liver and spleen only at 1 hours and day 1 and not at day 3 and day 7 (Figure 8).

### Effect of *S. typhimurium* A1-R on organ weight and histology

**Figure 9:**
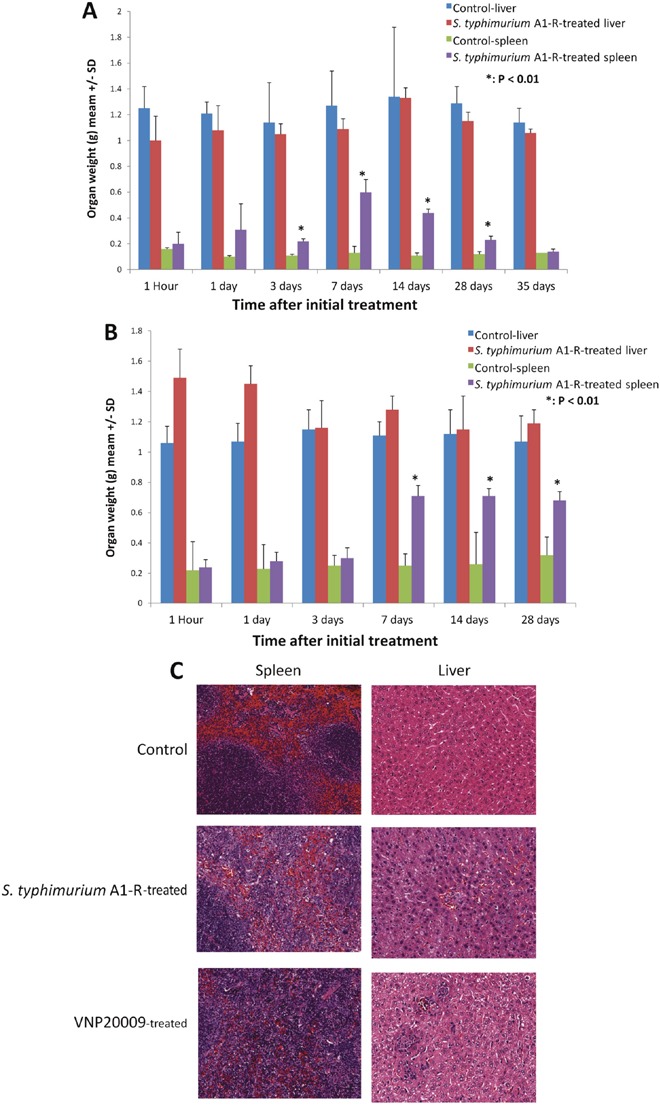
Toxicity of *S. typhimurium* A1-R on organ weight and *S. typhimurium* A1-R and *S. typhimurium* VNP20009 on organ histology **(A)** Non-tumor-bearing BALB/c mice were treated with 5 × 10^5^ CFU/100 μl PBS/mouse. Liver and spleen were removed, collected and weighed at different time points indicated above. N = 6 at each time point. **(B)** BALB/c mice bearing CT26 tumors with a tumor volume of (100 mm^3^) were treated with 5 × 10^5^ CFU *S. typhimurium* A1-R 100 μl PBS/mouse. Liver and spleen were removed, collected and weighed at different time points indicated above. N = 6. **(C)** Hematoxylin and eosin (H&E)-stained sections of spleens and livers of control, *S. typhimurium* A1-R-treated and VNP20009-treated non-tumor-bearing mice.

Each mouse received a single intravenous injection of *S. typhimurium* A1-R (5 × 10^5^ CFU). In non-tumor-bearing mice, only the spleen significantly increased in weight after treatment with *S. typhimurium* A1-R (5 × 10^5^ CFU) beginning at day 3 (Figure 9A).

In female BALB/c mice bearing subcutaneous CT26 mouse colon tumors, *S. typhimurium* A1-R (5 × 10^5^ CFU) treatment also caused the spleen to gain significant weight beginning at day 7, compared to control mice (Figure 9B).

Histopathologic analysis of liver and spleen at day 3 post-treatment of *S. typhimurium* A1-R or VNP20009 showed pathological changes in both organs (Figure 9C).

### Efficacy of *S. typhimurium* A1-R and VNP20009 on the CT26 colon tumor in BALB/c mice

**Figure 10:**
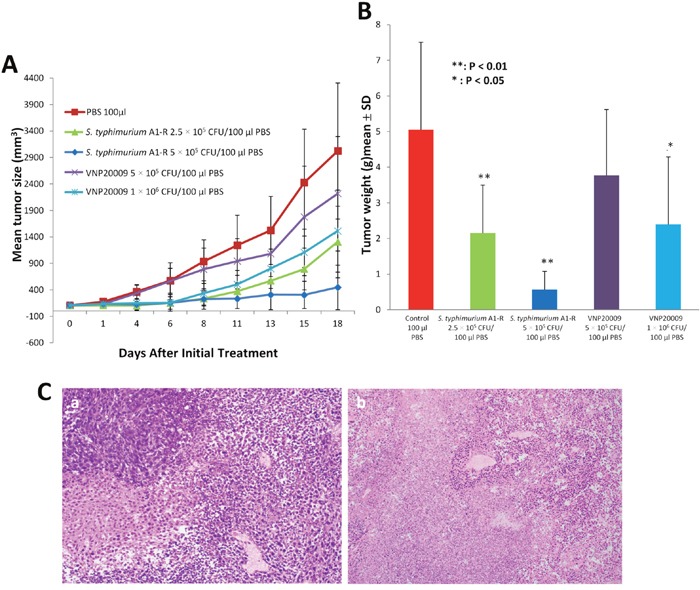
Antitumor efficacy of *S. typhimurium* and VNP20009 CT26 cells (2 × 10^6^/100 μl/mouse) were injected into the right flank of BALB/c mice. When the average tumor volume reached approximately 100 mm^3^, *S. typhimurium* A1-R (2.5 or 5 × 10^5^ CFU) or VNP20009 (5 or 10 × 10^5^ CFU) were injected into the tail vein once a week for three times. Primary tumor sizes were measured with a caliper. **(A)** Tumor growth curves comparing *S. typhimurium* A1-R or *S. typhimurium* VNP at both doses. **(B)** Tumor weight in *S. typhimurium* A1-R- and *S. typhimurium* VNP20009-treated mice, compared to control mice (*p<0.05; **p<0.01) at day 19 after treatment. n = 5 mice for each group. **(C)** Tumor histology shown in hematoxylin and eosin (H&E)-stained sections. **(a)** Tumor treated with VNP20009; **(b)** tumor treated with *S. typhimurium* A1-R.

*S. typhimurium* A1-R reduced tumor growth to a greater extent than VNP20009 (*p*<0.05). *S. typhimurium* A1-R at 5 × 10^5^ CFU arrested tumor growth. VNP20009 at 1 × 10^6^ CFU and *S. typhimurium* A1-R at 2.5 × 10^5^ CFU both slowed tumor growth to a similar extent, but could not arrest tumor growth (Figure 10A). VNP20009 at 1 × 10^6^ CFU caused extensive mouse body weight loss, unlike the effective 5 × 10^5^ CFU dose of *S. typhimurium* A1-R (Figure 4).

Tumor weight was measured on day 19. *S. typhimurium* A1-R at 2.5 × 10^5^ CFU (p<0.05) and 5 × 10^5^ CFU (p<0.01) decreased tumor weight. VNP20009 at 1 × 10^6^ CFU inhibited tumor weight (p<0.05), but VNP20009 at 5 × 10^5^ CFU did not inhibit tumor weight (Figure 10B). VNP20009 at 1 × 10^6^ CFU caused severed body weight loss, as mentioned above (Figure 4). Both *S. typhimurium* A1-R and VNP20009, treated tumor contained viable nuclei, small areas of necrosis, with accompanying acute inflammation.

Previously-developed concepts and strategies of highly-selective tumor targeting can take advantage of molecular targeting of tumors, including tissue-selective therapy which focuses on unique differences between normal and tumor tissues [33–38].

## MATERIALS AND METHODS

### Cell lines and other reagents

Murine CT26 colon cancer cells were obtained from ATCC (The American Type Culture Collection) and grown in RPMI 1640 Medium (Invitrogen, Carlsbad, CA) supplemented with 10% fetal bovine serum (FBS; HyClone, Logan City, UT) at 37°C with 5% CO_2_.

### Mice

Athymic *nu/nu* nude mice (AntiCancer Inc., San Diego, CA), 4–6 weeks old, were used in this study. Animals were housed in a barrier facility on a high efficacy particulate arrestance (HEPA)-filtered rack under standard conditions of 12-hour light/dark cycles. The animals were fed an autoclaved laboratory rodent diet. All mouse surgical procedures and imaging were performed with the animals anesthetized by subcutaneous injection of a ketamine mixture (0.02 ml solution of 20 mg/kg ketamine, 15.2 mg/kg xylazine, and 0.48 mg/kg acepromazine maleate). The response of animals during surgery was monitored to ensure adequate depth of anesthesia. The animals were observed on a daily basis and humanely sacrificed by CO_2_ inhalation if they met the following humane endpoint criteria: severe tumor burden (more than 20 mm in diameter), prostration, significant body weight loss, difficulty breathing, rotational motion and body temperature drop. All animal studies were conducted in accordance with the principles and procedures outlined in the National Institutes of Health Guide for the Care and Use of Animals under Assurance Number A3873-1.

### Preparation of *S. typhimurium* A1-R

GFP-expressing *S. typhimurium* A1-R bacteria (AntiCancer Inc.,) were grown overnight on LB medium (Fisher Sci., Hanover Park, IL, USA) and then diluted 1:10 in LB medium. Bacteria were harvested at late-log phase, washed with PBS, and then diluted in PBS [8, 11, 12].

### Comparison of OD_600_ and colony counting to quantify bacteria

GFP-expressing *S. typhimurium* A1-R were grown overnight in LB medium and then diluted 1:10 in LB medium. Bacteria were harvested at late-log phase, washed with PBS, and then serial diluted 10-fold for six times in PBS. OD_600_ measurements were performed on a spectrophotometer. Dilutions of bacteria were plated on LB agar and incubated at 37°C overnight. Colonies were counted under fluorescence microscopy to determine colonr forming units (CFU/ml) at each time point.

### Tumor inoculation

Murine CT26 colon cancer cells (2×10^6^) were injected subcutaneously into the right flank of BALB/c mice. Tumor volume was calculated as length × width^2^ × 0.5. In general, 10–14 days were required for tumors to reach the target size of 100 mm^3^ for treatment initation.

### Dose-tolerance experiments

The dose-tolerance relationship was examined in BALB/c mice for a single bolus or multiple doses once weekly for 3 consecutive weeks of *S. typhimurium* A1-R or VNP20009. Animal weights were compared with day 0 (first day of treatment administration) to determine percentage weight change. Mice were sacrificed at either 25% weight loss from day 0 or at the end of the observation period, which ever came first.

After administration of *S. typhimurium* A1-R or VNP20009 or control treatments, mice were closely monitored and weighed daily for 8 days, then at day 10 and 12, and twice weekly until the end of the observation period. At this stage, the animals were euthanized by CO_2_ inhalation.

### Biodistribution of *S. typhimurium* A1-R-GFP

At various time points after injection of *S. typhimurium* A1-R, mice were sacrificed by CO_2_ narcosis at 1 h, 1 day, 3 days, 7 days, 14 days and 28 days post-injection, and their blood (0.1 ml), brain, heart, lungs, liver, spleen, kidneys, muscle, bone, small and large intestines, and tumor were harvested. Tissues were removed, weighed, and homogenized in phosphate buffered saline (PBS), at 2 ml per 200 mg of tissue using an homogenizer (Fisher Scientific, USA) at a speed of ˜24,000 rpm for 30-60 s. The resulting suspension was serial diluted by 10× dilutions in PBS and carefully spread onto LB agar. Blood was obtained from intracardiac puncture and mixed with 9 volumes of PBS, after which a 100 μl suspension was spread on LB agar plates. LB agar plates were incubated for 18 h at 37°C. Following incubation, plates were removed from the incubator and colonies were counted. At least three mice were used for each time point. *S. typhimurium* A1-R-GFP colonies were visualized with an Olympus OV100 Small Imaging System with a CCD camera [39].

### Histological analysis

Fresh tumor and organ samples were fixed in 10% formalin and embedded in paraffin before sectioning and staining. Tissue sections (5 μm) were deparaffinized in xylene and rehydrated in an ethanol series. Hematoxylin and eosin (H &E) staining was performed according to standard protocols. Histological examination was performed with a BHS System Microscope (Olympus Corporation, Tokyo, Japan). Images were acquired with INFINITY ANALYZE software (Lumenera Corporation, Ottawa, Canada) [28].

### Anti-tumor efficacy

BALB/c mice bearing CT26 murine colon carcinoma xenografts were administered *S. typhimurium* A1-R or VNP20009 (i.v) with weekly doses indicated in Figure 10. Mice were monitored and weighed as described in the toxicity studies. Tumors were measured twice weekly using calipers along the x and y plane of the tumor. Tumor volume was determined using the formula volume = a x (b^2^)/2, where a is the largest diameter and b is the smallest diameter. Antitumor assessment of treatments was determined by reduction in tumor growth rate compared with controls.

### Statistical analysis

All statistical analyses were performed using SYSTAT 12.0 (SYSTAT, Inc., Chicago, IL, USA). The experimental data are expressed as the mean ± SD. The two-tailed Student's t-test was used for statistical analysis, with α equal to 0.05. P<0.05 was considered statistically significant.

## CONCLUSION

*S. typhimurium* A1-R has a similar toxicity pattern as VNP20009 which was previously determined to be safe in a Phase I clinical trial. However, *S. typhimurium* A1-R had significantly greater tumor targeting and anti-tumor efficacy than VNP20009. The results of the present study, therefore, indicate the promising clinical potential of *S. typhimurium* A1-R.

Bacterial therapy offers many advantages over conventional chemotherapy, including, but not limited to tumor-targeting, ability to deliver therapeutic cargoes, does not depend entirely on the tumor vascular system to reach the tumor since bacteria are motile, can stimulate the immune system against the tumor, can serve as adjuvant treatment after surgery to eliminate residual disease and can enhance chemotherapy by decoying quiescent drug-resistant cancer cells in tumors to attempt to cycle, thereby becoming sensitive to chemotherapy [4].

With regard to bacterial labeling, small molecular fluorescent probes or radionuclides may offer advantages over GFP to label *S. typhimurium* A1-R regarding translation to the clinic [40, 41]. Whether *S. typhimurium* A1-R can pass the blood-brain barrier is still an open question. Although, *S. typhimurium* A1-R was found at early time-points in the brain in the present study, it is possible that bacteria were actually from brain blood vessels in which the bacteria have not passed to the brain tissue itself. On the other hand, *S. typhimurium* A1-R prevented brain metastasis in a breast cancer mouse model [14]. It is not clear if the bacteria had to cross the blood-brain barrier for this anti-brain-metastasis efficacy. Future experiments will address this point.

## DEDICATION

This paper is dedicated to the memory of A. R. Moossa, M.D., and Sun Lee, M.D.
